# SHARED EXPERIENCES OF MANAGING HEART FAILURE AND MULTIMORBIDITY: A QUALITATIVE ANALYSIS

**DOI:** 10.1093/geroni/igad104.2888

**Published:** 2023-12-21

**Authors:** Katie Nelson, Chitchanok Benjasirisan, Arum Lim, Lyndsay DeGroot, Sarah Badawi, Cheryl Dennison Himmelfarb, Patricia Davidson, Binu Koirala

**Affiliations:** Johns Hopkins Center for Indigenous Health, Baltimore, Maryland, United States; Johns Hopkins University, Baltimore, Maryland, United States; Johns Hopkins University, Baltimore, Maryland, United States; Johns Hopkins University School of Nursing, Baltimore, Maryland, United States; Johns Hopkins University School of Nursing, Baltimore, Maryland, United States; Johns Hopkins University, Baltimore, Maryland, United States; University of Wollongong, Wollongong, New South Wales, Australia; Johns Hopkins University School of Nursing, Baltimore, Maryland, United States

## Abstract

Heart failure affects more than 6 million people nationally; most are older adults who have multimorbidity. Navigating symptom management and self-care maintenance can be challenging given heart failure’s non-linear trajectory. Therefore, identifying shared priorities is important for guiding steps to enhance care provision. Our study aimed to elicit experiences and perspectives regarding heart failure and multimorbidity management among patients, caregivers, and healthcare workers via semi-structured interviews. We used purposive and convenience sampling to recruit patients 50 years and older living with heart failure and comorbidities, family caregivers, and interdisciplinary healthcare workers from an academic medical center in the Mid-Atlantic region of the U.S. Participants were asked about disease trajectory, recognizing/treating symptoms, and service utilization. We used inductive thematic analysis and presented key findings at virtual co-design events, whereby participants ranked priorities to determine next steps. Forty-six interviews were conducted: 24 patients (mean ± SD: 70.1 ± 9.16 years; 46% female), 7 family caregivers (63.6 ± 14.18 years; 57% female), and 15 healthcare workers (35.6 ± 10.04 years; 80% female). Four themes were emphasized across groups: 1) clear communication, 2) health education for patients and caregivers, 3) care coordination and follow-up post-discharge, and 4) provision of mental and emotional support. Patients, caregivers, and healthcare workers uniquely contribute to heart failure and multimorbidity management. All groups share priorities surrounding clear communication, health education and comprehensive support. These factors can be significant barriers or facilitators in terms of optimizing patients’ symptoms and quality of life.

